# The challenges and importance of structural variation detection in livestock

**DOI:** 10.3389/fgene.2014.00037

**Published:** 2014-02-18

**Authors:** Derek M. Bickhart, George E. Liu

**Affiliations:** ^1^Animal Improvement Programs Laboratory, United States Department of Agriculture–Agricultural Research ServiceBeltsville, MD, USA; ^2^Bovine Functional Genomics Laboratory, United States Department of Agriculture–Agricultural Research ServiceBeltsville, MD, USA

**Keywords:** CNVs, SV, livestock genomics, insertions and deletions (indels), olfaction, coat color genetics, antimicrobial peptides

## Abstract

Recent studies in humans and other model organisms have demonstrated that structural variants (SVs) comprise a substantial proportion of variation among individuals of each species. Many of these variants have been linked to debilitating diseases in humans, thereby cementing the importance of refining methods for their detection. Despite progress in the field, reliable detection of SVs still remains a problem even for human subjects. Many of the underlying problems that make SVs difficult to detect in humans are amplified in livestock species, whose lower quality genome assemblies and incomplete gene annotation can often give rise to false positive SV discoveries. Regardless of the challenges, SV detection is just as important for livestock researchers as it is for human researchers, given that several productive traits and diseases have been linked to copy number variations (CNVs) in cattle, sheep, and pig. Already, there is evidence that many beneficial SVs have been artificially selected in livestock such as a duplication of the agouti signaling protein gene that causes white coat color in sheep. In this review, we will list current SV and CNV discoveries in livestock and discuss the problems that hinder routine discovery and tracking of these polymorphisms. We will also discuss the impacts of selective breeding on CNV and SV frequencies and mention how SV genotyping could be used in the future to improve genetic selection.

## INTRODUCTION

### SV CLASSIFICATION

The post-genome era has revealed new classes of structural variants (SV) in Eukaryotic genomes that have eluded easy detection and characterization. Recognized classes of SVs include copy number variants (CNVs), segmental duplications (SDs), inversions and translocations (Feuk et al., 2006). SDs, also termed “low copy repeats,” are large continuous stretches of DNA that can be mapped to multiple locations on the genome and share >90% nucleotide similarity with each other (Bailey and Eichler, 2006). The higher frequencies of SDs within the human population suggest that they are shared duplications that have been fixed in the population rather than being recurrent structural mutations (Sharp et al., 2005; Bailey and Eichler, 2006). By contrast, CNVs are defined as duplications or deletions of genomic segments that range in size from 50 basepairs (bps) to megabasepairs (mbp) and vary among individuals of a species (Conrad et al., 2010). The two other SV categories, inversions and translocations, are relatively self-explanatory; being large-scale inversions of genomic sequence and large transfers of genomic DNA from one region of the genome to another, respectively.

SV types can be further classified as balanced (inversions and translocations) and unbalanced (CNVs and SDs) events based on their resulting copy number changes in the affected individual (Feuk et al., 2006). These two categories represent important distinctions, as the methods used to detect SVs are highly dependent on the resulting proportion of genomic sequence they create/remove. Among the unbalanced class of SVs, SDs, and CNVs often comprise a large proportion of the genome, ranging from 5.2% (SDs) to ~12% (CNVs) of the human reference genome (Bailey and Eichler, 2006; Redon et al., 2006; Levy et al., 2007). Detection of unbalanced events is often inferred from a loss or gain of genomic sequence (also called “read depth” or RD; Alkan et al., 2009; Sudmant et al., 2010) or array probe signal intensity (Lockwood et al., 2005; Wang et al., 2007) within the affected region as compared against the reference genome. Methods designed to identify unbalanced SVs from array and sequence data are more mature than methods focusing on balanced events given the need to identify sequence breakpoints in order to detect a balanced event [for a review see: (Alkan et al., 2011)]. Balanced SVs such as inversions and chromosomal translocations can impact organism phenotypes (Durkin et al., 2012) but remain particularly difficult to detect as *de novo* events, as they do not alter the copy number of involved genes. Inversions are virtually undetectable when using array-based discovery methods, leaving PCR (Liu et al., 1999) or sequencing (Tuzun et al., 2005) as the only viable methods of detection. Specialized sequencing methods that involve paired-end sequence data (called “read pair” or RP) have been developed to identify these mutations (Korbel et al., 2007); however, difficulty in validating these mutations experimentally prevents their reliable detection.

### SV FORMATION MECHANISMS

**FIGURE 1:**
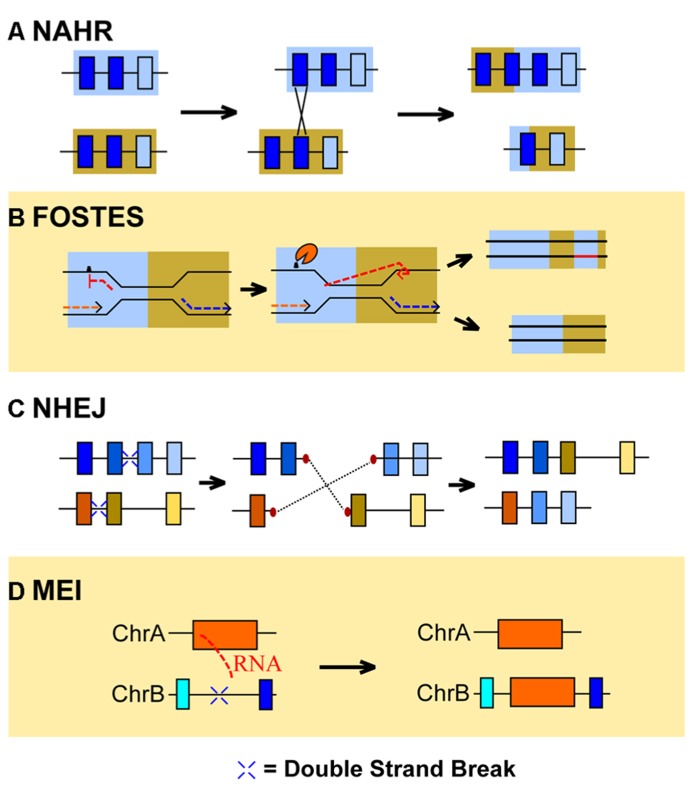
** The molecular mechanisms of CNV formation**. Currently identified mechanisms of copy number variation (CNV) formation include non-allelic homologous recombination (NAHR), fork stalling and template switching (FOSTES), non-homologous end-joining (NHEJ), and mobile element insertion (MEI). **(A)** NAHR generates CNVs when a genomic segment with high sequence similarity to another, non-allelic locus (blue boxes) recombines. The results of this recombination can generate a duplication of the similar locus on one chromosome, while removing the copy from the other. **(B)** FOSTES occurs when the DNA replication complex stalls due to DNA lesions or chemical modifications of the nucleoside bases (hatch mark) and the lagging strand of DNA (red dashed line) associates with a different region of the genome with high sequence similarity. The location of the association determines if a duplication (pictured) or deletion occurs. **(C)** Double stranded breaks in DNA sequence (blue crosses) prompt NHEJ associated proteins to repair and ligate DNA strands together. First, end-repair (red ovals) replaces lost nucleotides on the double strand break and DNA ligase associates broken DNA fragments together. If fragments from different chromosomes ligate together, duplications or deletions of sequence can occur. **(D)** Retrotransposition involves an RNA intermediate (red dashed lines) that is reverse transcribed into cDNA and is subsequently inserted into the genome, thereby causing a duplication of the original endogenous retrovirus.

One of the primary means by which CNVs form within the genome is due to a phenomenon called non-allelic homologous recombination (NAHR, **Figure 1**; Gu et al., 2008). NAHR often occurs during meiosis, where two regions that are not alleles of each other but share significant sequence homology cross-over due to a normal recombination event. In the case of unequal cross-over events caused by NAHR of different chromosomes, one sister chromosome can increase in size at the cost of another’s expense. NAHR of genomic segments on the same chromosome can cause deletions of segments due to circular intermediates (van Loon et al., 1994). CNVs are frequently found in close proximity to SD regions of the genome (Kim et al., 2008), suggesting that NAHR among SD repetitive regions can contribute to CNV formation in individuals.

In addition to the previously mentioned mechanism, NAHR, fork stalling and template switching (FOSTES), mobile element insertion (MEI), and non-homologous end-joining (NHEJ) have also been implicated in the formation of CNVs (Gu et al., 2008). FOSTES occurs when the DNA replication machinery pauses, the lagging strand dissociates from the polymerase holoenzyme and associates the lagging strand with another region of the genome before replication is restarted (Lee et al., 2007). The genomic segment that caused the stalling of the polymerase is therefore duplicated if the lagging strand hybridizes with a segment of DNA downstream of the problematic region. Pausing of the DNA replication machinery is common at certain nucleotide motifs and repetitive DNA sequences (Viguera et al., 2001); however, such events can also occur due to chemical changes in DNA structure such as DNA lesions or DNA alkylization (Minca and Kowalski, 2011). The fact that such an error occurs during DNA replication, suggests that CNVs generated by FOSTES only occur during the S phase of the cell cycle as a consequence of DNA repair mechanisms that require DNA replication. It should also be noted that the types of CNVs created through FOSTES are difficult to distinguish from those generated by micro-homology-mediated breakpoint-induced repair (MMBIR), a mechanism of end-joining that relies on small-scale homology of DNA sequence at the ends of double strand breaks (DSBs) of DNA (Zhang et al., 2009). For the purposes of this review, we refer to both FOSTES and MMBIR mechanisms as “FOSTES” unless direct evidence supporting MMBIR was identified in the literature.

Another mechanism of CNV formation is more closely correlated with DNA repair mechanisms. NHEJ is a DNA repair mechanism that is frequently initiated in response to DSBs in DNA sequence (van Gent and van der Burg, 2007). In NHEJ, DNA DSBs are identified, repaired and ligated together, oftentimes to different regions of the genome than they originated. An interesting characteristic of NHEJ mediated repair is that it is not dependent on the presence of SDs or repetitive regions, and can thereby occur in any genomic region susceptible to DSBs (Gu et al., 2008). An interesting consequence of the repair process is that a “scar” sequence of nucleotides is left at the repair site from the end-repair of the previous DSB fragments (Gu et al., 2008). NHEJ is more often associated with deletions (Inoue et al., 2002; Shaw and Lupski, 2005) and chromosomal translocations (Lieber et al., 2010); however, complicated DNA intermediates have been proposed as a method for duplications to occur through NHEJ as well (Lee et al., 2006). The final mechanism, MEI, is the subject of extensive review. Interested readers are encouraged to read the recent review by Treangen and Salzberg (2012).

### GENE DOSAGE EFFECTS CAUSED BY CNVs

Genic CNVs are predicted to influence organism phenotypes through several phenomena such as gene dosage, expression regulation changes and recessive allele exposure. Duplication and deletion of cis-regulatory elements have been shown to greatly influence phenotype, particularly when such CNVs influence developmental genes (Spielmann and Klopocki, 2013). Additionally, deletion of a normal allele in heterozygous carrier individuals has been shown to cause disease states by exposing the recessive allele (Boone et al., 2013). Recent experiments profiling the effects of CNVs on organism fitness have focused on gene dosage, which is a mechanism by which the alteration of gene copy number changes the expression profile of the gene. The balance hypothesis predicts that genes that code for multiple component protein complexes, or genes that are central to major regulatory networks are likely to be more sensitive to CNV-induced alterations in gene expression (Papp et al., 2003). The effects of gene dosage perturbation can be best illustrated by the frequent lethality that accompanies polyploidy in most mammalian species. Such large-scale imbalances in chromosome number, collectively termed “chromosome aneuploidy,” have been shown to directly influence the expression levels of genes on the variant chromosome (Muers, 2012) and are often lethal to the organism (Torres et al., 2008). Indeed, Schuster-Böckler et al. (2010) found that predicted dosage sensitive gene families were significantly underrepresented within CNV regions (CNVRs) in humans. Given that CNVs of dosage sensitive genes are poorly tolerated, it appears that specific regions of the genome are less likely to harbor SVs. Tests of this hypothesis through genome-wide profiling has suggested that only 3% of yeast genes are sensitive to haploinsufficiency (Deutschbauer et al., 2005). Within multi-cellular Eukaryotes, only 21% of detected *Drosophila* genic CNVs altered the expression levels of the impacted genes (Zhou et al., 2011). Such low percentages may be an underestimation of the impact of CNVs on gene expression, as CNVs involving cis- and trans-regulatory elements were not identified and assayed in these studies. Identifying regulatory elements that influence gene expression using computational methods is notoriously difficult (Ponting and Hardison, 2011), so true estimates of the functional impacts of CNVs on gene expression may need a large library of experimentally determined regulatory element binding sites (Consortium, 2012) in addition to validation experiments to confirm differences.

## THE EVOLUTIONARY AND FUNCTIONAL IMPACTS OF CNVs IN LIVESTOCK

### ARTIFICIAL SELECTION IN THE AGE OF GENOMICS

Livestock species have a nuanced history of evolutionary influences resulting from selection pressures from the environment and their handlers as well. Charles Darwin noted in “The Origin of Species” that the diversity of pigeon species was derived from the selection of unique external phenotypes that arose from repeated breedings (Darwin, 1859). As such, external phenotypes remain the clearest result of artificial selection in our domestic species of livestock, though such phenotypes often evade easy classification on the molecular level. Recent advances in genomics have allowed the creation of new genotyping tools that allow breeders to identify specific genomic segments that have transitioned from parents to progeny for a more precise artificial selection of traits. The development of the Illumina BovineSNP50 genotyping array (Matukumalli et al., 2009) has revolutionized genomic selection in cattle by allowing inexpensive genotyping, which in turn can be used to associate genetic segments with quantitative traits (VanRaden, 2008). This genotyping array allows breeders to assess the competence of young bulls at earlier ages, therefore the generation interval for dairy cattle breeding has shortened substantially (Hutchison et al., accepted by Journal of Dairy Science). Extensive use of the BovineSNP50 array in the dairy cattle genotyping industry and the availability of that data to researchers has resulted in a larger number of array-based CNV studies in cattle than in other livestock species (Fadista et al., 2010; Hou et al., 2011, 2012a, b; Liu et al., 2011). The results of such studies have revealed an increasingly complex landscape of CNV within the cattle genome, raising questions as to how CNVs impact productive traits and if such CNVs are being selected from the population for all livestock species.

### GENE FAMILY DUPLICATION AND EVOLUTION

The fundamental basis for gene family expansion and contraction appears to be tightly linked to SVs that are exposed to selective pressures. The current evolutionary model that best explains why paralogous gene families appear to be conserved within Eukaryotic genomes is the “birth and death” model (Nei and Rooney, 2005). In this model, gene families expand and contract – likely due to NAHR, FOSTES, or NHEJ mechanisms – and are subjected to either diversifying or stabilizing selection (Nei and Rooney, 2005). A clear example of the predictive power of this model has been within the olfactory receptor (OR) gene families, where overdominance (a product of diversifying selection) has been cited as a primary means by which OR paralogs have been conserved within a species’ genome (Alonso et al., 2008; Young et al., 2008). Given the combinatorial nature of OR-odorant associations (Malnic et al., 1999), it is a benefit for the host organism to maintain a broad selection of ORs so as to detect a wide range of odorant molecules (see Olfaction as a Result of CNVs). Under similar selective pressures, the lysozyme gene family appears to have underwent an expansion within mammalian species (Grobler et al., 1994). Sequence homology searches have revealed 18 lysozyme family members identified within the cattle genome (Irwin et al., 2011). Diversifying selection in the presence of evolving bacterial microflora appears to be the reason for this expansion, as a study has identified several bacterial species that have developed a resistance to bovine gastric lysozyme (Domínguez-Bello et al., 2004).

One must consider the fact that CNVs tend to occur within certain hotspots more than in others (Kim et al., 2008; Gokcumen et al., 2011). Indeed, it appears that the noted expansion of the OR gene families occur due to their proximity to SD regions in the genome (Young et al., 2008). Interestingly, the proximity of lysozyme gene family members to OR gene family members appears to have driven their expansion as well (Irwin et al., 2011), most likely due to the later gene family’s proximity to SD regions. This represents a unique evolutionary strategy: proximity to regions that are prone to frequent duplications tends to result in the expansion of nearby gene family members and can promote the diversification of gene family function. Several CNV studies in Cattle have identified similar associations of SDs with duplications and deletions of genic regions (Hou et al., 2011; Cicconardi et al., 2013). Identification of additional genes that are subject to SD-mediated duplication will allow for better detection of emergent gene families within the genome.

### PHENOTYPES AFFECTING PIGMENTATION AND COAT COLOR

**FIGURE 2:**
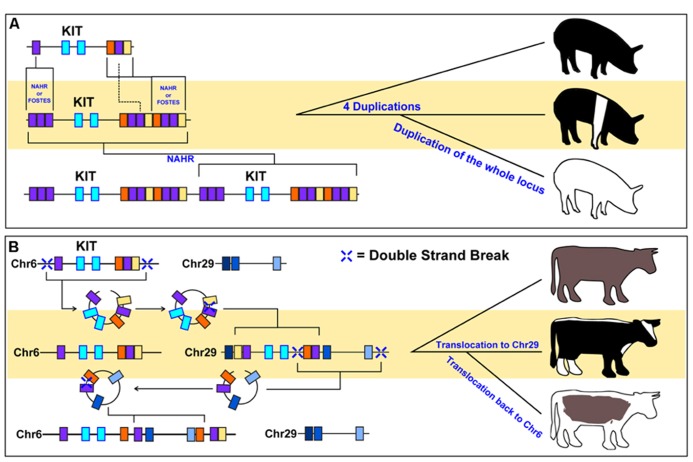
** Examples of phenotypes caused by CNV formation**. Selection of different CNVs within animal populations can leave evidence as to the evolutionary origins of the phenotypes they grant. **(A)** Figure adapted from Rubin et al. (2012). Duplications of regulatory elements upstream and downstream of the KIT gene locus (colored boxes) resulted in a belted phenotype in pigs. Subsequent duplication of this altered KIT gene locus, in addition to a splice site variant that excludes exon17 (not shown), results in the dominant white phenotype. **(B)** Figure adapted from Durkin et al. (2012). Translocation of the KIT locus, in addition to surrounding regulatory genomic segments, has resulted in distinct coloration phenotypes in cattle. It was discovered that the color-sided phenotypes in Belgian Blue (middle) and Brown Swiss (bottom) cattle were achieved by two translocations of the KIT locus to different cattle chromosomes. The rearrangement of surrounding genomic segments (colored boxes) near the KIT locus at each translocation point suggested that circular intermediates were involved in the movement of this locus.

The coat colors of sheep and pigs represent clear examples of CNVs selected by artificial selection (**Table 1**). White coats are preferred in sheep as the resulting white hair forms wool that can be easily dyed to alternative colors. The expression profile of the agouti signaling protein (ASIP) gene is substantially increased in the hair follicles of sheep that contain a duplicate copy of the gene that is directly downstream of the ITCH promoter (Norris and Whan, 2008; Fontanesi et al., 2009, 2011). This modified ASIP duplication has been linked to the typical white coat color associated with domestic sheep species. A strong signature of selection originates from the ASIP and KIT loci in sheep (Kijas et al., 2012), providing corroborating evidence of strong artificial selection for this duplicated allele. Similarly, a duplication of the ASIP gene in goats was found to promote white coat color as well (Fontanesi et al., 2009). Cross-breed comparisons in pigs have revealed at least four detectable duplications within the KIT locus that could represent causal mutations for both the belted and dominant white phenotypes (Rubin et al., 2012). White coats in pigs are easier to remove at slaughter (Cannon et al., 1996) and are more appealing to consumers of skin-on pork products. Duplication of an allele of the KIT gene (Giuffra et al., 2002; Fadista et al., 2008; Rothschild and Ruvinsky, 2011) with a splice site variant that excludes exon17 (Marklund et al., 1998) results in a dominant white phenotype. A patchy white coat phenotype results from the KIT duplication alone (Rothschild and Ruvinsky, 2011), suggesting that exon17 has substantial phenotypic influence when duplicated as part of the gene. The duplications of KIT appear to have resulted from NAHR of two LINEs flanking the gene (Giuffra et al., 2002), again highlighting the role of recombination in CNV formation (**Figure 2A**).

**Table 1:** Genes associated with external phenotypes that are influenced by structural variants in livestock.

Gene	Phenotype	Animal	Description	References
KIT	Color-sidedness	Cattle	Circular intermediate translocation of KIT gene locus from BTA6 to BTA29, with a subsequent translocation back to BTA6	Durkin et al. (2012)
	Belt, patch and dominant white	Pig	Tandem duplications of KIT locus exons on Chr8	Rubin et al. (2012)
ASIP	White coat	Sheep	Duplicate of the gene under the control of the ITCH promoter.	Fontanesi et al. (2011), Norris and Whan (2008)
	White coat	Goat	Duplication of the ASIP gene locus.	Fontanesi et al. (2009)
PRLR and SPEF2	Late feathering	Chicken	A duplication of the K locus, which contains the SPEF2 and PRLR genes along with an endogenous retrovirus insertion	Elferink et al. (2008), Wang et al. (2010)
EDN3	Excessive black pigmentation	Chicken	A 130 kb duplication of a locus containing the EDN3 gene	Shinomiya et al. (2012)
SOX5	Pea-comb		A duplication of the first intron of the SOX5 gene	Wright et al. (2009)

Similar to what has been found in pigs and sheep, cattle coat color has primarily been determined by inheritance of different alleles of the KIT, ASIP, TYRP1, and MC1R genes (Gutiérrez-Gil et al., 2007; Schmutz, 2012). Recently, a number of studies indicate a chromosomal translocation (and subsequent duplication) of the KIT gene in several cattle coat phenotypes (**Figure 2B**; Durkin et al., 2012; Brenig et al., 2013). Durkin et al. (2012) found that a 492 kbp segment of BTA6 containing the KIT gene was translocated to BTA29 in several Brown Swiss and Belgian Blue cattle via two circular intermediate steps, and a reshuffling of the order of genes on the transferred segment. This phenotype was generated in two steps, with the first step involving the insertion of the BTA6 KIT locus to BTA29 in Belgian Blue cattle through a mechanism predicted to be micro-homology mediated end-joining. Subsequent breeding resulted in animals that regained the wild-type copy of the KIT gene on BTA6, but still had the translocated copy of KIT on BTA29. The second step involved a translocation of part of the new BTA29-BTA6 fusion locus back to the original BTA6 wild-type locus via NAHR. While the translocation and incorporation of DNA circular intermediates has been well characterized in bacterial integron dynamics (Domingues et al., 2012), this is one of the first instances where a similar mechanism was detected in a mammalian species, let alone a complex double-translocation event. The implications of this new allele have extended beyond Brown Swiss and Belgian Blue cattle as White Galloway and White Park cattle were found to carry this allele (Brenig et al., 2013). Surprisingly, the effects of the modified KIT locus in these cattle result in mottled markings rather than color-sidedness, suggesting that the extent by which modification of KIT can influence coat color has not been fully explored (Brenig et al., 2013).

Work on CNVs in chickens has also identified several important genes that have been modified by CNVs, resulting in several clear phenotypic changes. Perhaps one of the most recognizable chicken phenotypes attributable to CNVs is the peacomb phenotype. Characterized by a reduction in the size of the combs of male and female chickens, the peacomb phenotype is attributed to a duplication of the first intron of the SOX5 gene (Wright et al., 2009). Chicken do not dissipate internal body heat through sweating and must rely on their combs for heat regulation (Sturkie, 1965). Wright et al. (2009) thereby postulate that the peacomb phenotype is an advantage to chicken in colder climates by reducing heat-loss. A partial duplication of the PRLR and SPEF2 genes has been linked to a late feathering phenotype in several breeds of chicken (Elferink et al., 2008; Wang et al., 2010). The Chinese silky breed of chicken is known for its curled, abnormal feathers and black tissues. Abnormal migration of melanoblasts into the epithelium – similar to the migration of fibroblasts – is the cause of the excessive black pigmentation of internal tissues (Faraco et al., 2001). A study by Shinomiya et al. (2012) identified a duplication of the endothelin 3 gene as the potential source of this phenotype, as endothelin 3 transcription was found to be heightened in during neural crest migration in Silky chickens. A study in transgenic mice found that overexpression of the EDN3 gene at early stages of embryonic growth produced hyperpigmentation (Garcia et al., 2008), confirming the results of Shinomiya et al. (2012) This duplication has been confirmed by a recent CNV study using next generation sequencing (NGS) data derived from a silky chicken (Fan et al., 2013).

### OLFACTION AS A RESULT OF CNVs

Olfactory receptor proteins expressed by cells within olfactory organs in vertebrates allow for the detection of volatile chemicals in the environment. Rather than being specific for a single type of odorant, ORs have been shown to have varying affinities for a wide range of molecules (Malnic et al., 1999). This property allows an organism to detect many more odorant molecules than the number of OR genes in its genome would imply. Recent work on Artiodactyla CNVs has revealed extensive variation in OR gene copy number in different livestock species (Nguyen et al., 2012; Lee et al., 2013), suggesting that the evolutionary basis of odorant detection may have been due to selection on gene duplicates of this family. Olfaction is especially important to pigs, as their sense of smell is important in foraging (Groenen et al., 2012). A recent study identified OR locus variation that could be attributed to pig breeds derived from different geographic regions (Paudel et al., 2013). The authors suggest that such variation could be attributed to selective pressures resulting from the different volatile chemicals that pig breeds may encounter from the different regions; however, a prior report on OR gene diversity in pigs found that OR gene clusters appear to be conserved across species and that OR genes appear to be duplicated within such clusters (Nguyen et al., 2012). It is interesting to note that a recent assembly of a Tibetan wild boar found a 40% reduction in the number of OR genes in the breed when compared against the current pig assembly (Li et al., 2013). The authors suggested that the low barometric pressure and lower humidity associated with high altitude environments resulted in a reduction of selection for OR genes in Tibetan boars compared to the Duroc, domestic pig (Li et al., 2013). This supports the findings of Paudel et al. (2013) and suggests that the OR gene family evolution may subject to more selective pressures than originally believed.

### INNATE AND ADAPTIVE IMMUNITY

Diversifying selection may influence the copy number of several innate immunity gene families within livestock species (**Table 2**). As mentioned previously, subsequent selection on duplicated gene paralogs can create or destroy new gene families with related or similar function (Nei and Rooney, 2005). There is sufficient evidence that many genes belonging to the innate and adaptive immune system are copy number variable within vertebrate species, particularly within the major histocompatibility complex (MHC) genes (Ohtsuka et al., 2008; Balakrishnan et al., 2010). Indeed, many livestock CNV surveys report substantial immune gene enrichment within CNV regions (Bickhart et al., 2012; Choi et al., 2013). SD maps in cattle and dog have already identified a correlation between SDs and the placement of immune system related genes (Liu et al., 2009; Nicholas et al., 2009), which suggests that duplications of these genes are likely frequent events caused by NAHR, or are already fixed in the populations within the SDs themselves. We will attempt to divide our discussion on immune system related genes by class; however, we will make the distinction between genes related to the adaptive and innate immune systems where appropriate.

**Table 2:** Immunity-related genes influenced by structural variants.

Gene family	Gene	Description	References
MHC class I	ENSGALT00000028239, ENSGALT00000004115	Deletion of MHC class I antigen-presenting proteins in chicken was associated with Marek’s disease resistance	Luo et al. (2012)
MHC class II	CIITA	CIITA, a trans-activator of MHC II, was duplicated in cattle with nematode resistance	Liu et al. (2011)
Antimicrobial peptides	LAP, TAP, BSP30A	Cattle-specific, β-defensin family, antimicrobial peptides (AMPs) with high copy number	Bickhart et al. (2012)
	PGN3, CATHL4	Cathelicidin family AMPs from pig and cattle, respectively, with high degrees of copy number variation	Paudel et al. (2013), Bickhart et al. (2012)
Endogenous retroviruses	enJSRV	A variant of the endogenous Jaagsiekte sheep retrovirus that is highly duplicated, protects individuals from the exogenous virus, which causes pulmonary adenocarcinoma	Viginier et al. (2012)
T cell receptors	WC1	CNVs of the cattle-specific WC1 gene have been identified	Liu et al. (2010), Chen et al. (2012)

Antimicrobial peptides (AMPs) represent a class of copy number variable genes within livestock species that function as part of the innate immune response to pathogens. The β-defensin class of AMPs appears to be copy number variable in several livestock species, but most notably in cattle (Liu et al., 2010, 2011). As opposed to α-defensins produced by polymorphonuclear leukocytes and paneth cells in the intestines, β-defensins are typically produced by epithelial tissues in order to defend against bacterial and fungal pathogens [for a review see: (Weinberg et al., 2012)]. Using extensive genetic mapping, Bakar et al. (2009) identified a CNV locus containing seven β-defensins that was duplicated and inverted approximately five megabases away on human chromosome 8. Evidence has emerged that this CNVR may be under positive selection in human populations in Asia, given the higher frequency of one duplicated region compared against other geographic locations (Hardwick et al., 2011). Similarly, population-scale genotyping of β-defensin gene member copy number in livestock species may reveal differential copy number preferences based on the geographic regions inhabited by each subpopulation. Already, studies in cattle (Liu et al., 2009, 2010; Bickhart et al., 2012), suggest that β-defensins are a highly duplicated gene family with recognizable intra-individual fluctuations of copy number. Recently, cattle-specific β-defensin genes have been identified as highly copy number variable within a CNV survey using sequence data (Bickhart et al., 2012). The lingual antimicrobial peptide (LAP) and tracheal antimicrobial peptide (TAP) genes share a high degree of sequence homology with other β-defensins, but are AMPs exclusive to cattle (Luenser and Ludwig, 2004). Additionally, the BSP30A gene, which is an important salivary AMP, was found to be highly copy number variable within cattle of different breeds (Bickhart et al., 2012). Finally, cathelicidin-type AMPs such as PGN3 (Paudel et al., 2013) and CATHL4 (Bickhart et al., 2012) have been identified as highly variable among pig and cattle individuals, respectively. With respect to the latter gene, CATHL4 was found to have a 2–4 fold higher expression in Nelore cattle neutrophils (Flores, 2011) possibly owing to its higher copy number in that breed. It is likely that members of these AMP gene families have proliferated under overdominance selection caused by ever-evolving bacterial species that are consumed while grazing. CNV surveys in other livestock species are likely to reveal other AMP families that are copy number variable and could offer resistance to bacteria in different geographic regions.

MHC gene family members have been frequently found to be copy number variable in livestock species; however, CNVs of the different classes of MHC genes need to be interpreted carefully by the community. MHC genes encode for receptor proteins that fall within two classes, labeled class I and II respectively, [for a review see: (Neefjes et al., 2011)]. Class I receptors are expressed ubiquitously and present small polypeptides resulting from proteaosome cleavage within the cell to circulating natural killer (NK) T cells. This is a way to detect intra-cellular pathogens as protein “garbage” resulting from foreign bodies inside the cell can be detected by the NK cells when presented on MHC class I receptors. Marek’s disease resistance was associated with CNVs of the MHC class I receptors in chicken (Luo et al., 2013), suggesting that duplication of this class of genes putatively influences the ability of NK cells to detect infected somatic cells. Whereas MHC class I receptors are present on all nucleated cells and make up a component of innate immunity, MHC class II receptors are present only on dedicated antigen-presenting cells and form an important first step in the adaptive immune system (Neefjes et al., 2011). A duplication of the CIITA gene, which encodes a trans-activator of the MHC class II receptor, was found in cattle that had resistance to ingested nematodes (Liu et al., 2011). In addition to this example, studies on the loss of copy number of MHC class II genes within other species have revealed increased susceptibility of that species to pathogens and cancers, such as the Tasmanian devil facial tumor epidemic (Cheng et al., 2012). This should serve as a warning to all animal breeders, as a loss of diversity at this locus due to improperly managed selective breeding or imposed population bottlenecks could increase the susceptibility of their herds to epidemics (Eimes et al., 2011).

Several other classes of immunity related gene families have been identified as copy number variable in livestock species. Expansion and contraction of the workshop class I (WC1) gene family has been identified in cattle (Liu et al., 2010; Bickhart et al., 2012; Chen et al., 2012). WC1 genes are unique to the cattle, sheep, and pig genomes, and encode pattern recognition receptors expressed on γδT cells (Herzig and Baldwin, 2009). A highly duplicated endogenous retrovirus that granted immunity to a similar, related virus that causes pulmonary adenocarcinoma was recently found in sheep (Viginier et al., 2012). This represents an unusual case of innate immunity caused by a domesticated, former pathogen of the species. Given the ubiquity of immunity related genes that coincide with CNVs, there are likely many more immunity traits that are influenced by CNVs. However, the complexity of immune system gene pathways and the paucity of expression studies on these genes make the estimation of the functional impacts of such relationships difficult.

## CHALLENGES TO SV DETECTION IN LIVESTOCK

### REFERENCE GENOME ASSEMBLY PROBLEMS

Genomics researchers have often relied on a reference genome assembly to assign variants to their proper genomic context. Additionally, genome assemblies also reduce the computational time involved in the analysis of sequence data by allowing for the alignment of reads against a comparative sequence of DNA. The reader would be astute to note that most of the livestock species mentioned in this review currently have reference genome assemblies available to the public. Specifically, reference assemblies exist for cattle (Elsik et al., 2009; Zimin et al., 2009), chicken (International Chicken Genome Sequencing Consortium, 2004), sheep (International Sheep Genomics Consortium et al., 2010), and pigs (Fang et al., 2012; Groenen et al., 2012) with a goat sequencing project only recently reaching a draft stage (Dong et al., 2013). Errors in a reference genome assembly can often result in misinterpretations of the underlying sequence of a sample, particularly when SVs are the focus for detection. It was found that over 14 megabases of predicted SDs in the galgal3 reference assembly were actually assembly errors (Kelley and Salzberg, 2010). Likewise, 39 megabases of SDs in the Btau4.2 cattle reference assembly were likely due to misassemblies and were not true SD regions (Zimin et al., 2012). The relatively lower quality of reference genomes produced for livestock species substantially increases the amount of false positives produced in the detection of SVs. Assembly gaps and unplaced contigs represent substantial difficulties for SV detection as well. A survey of CNVs on the earlier Btau4.0 cattle reference assembly identified 52 candidate CNVs within unplaced contigs (Liu et al., 2010). Although these CNVs comprised 41.1% of the total number of copy number variable nucleotides discovered, Liu et al. (2010) cautioned against interpreting these results liberally, given the uncertain nature of the unplaced contigs.

Reference genomes are still noticeably absent for several livestock species, thereby restricting the types of analysis that can be performed. Generating a reference assembly is not a trivial matter as it requires extensive computational logistics (Zhang et al., 2011) and technical expertise (Nagarajan and Pop, 2013). International consortia are currently working on reference assemblies for *Bos taurus indicus* and *Bubalis bubalus* (VanTassell, C.P., personal communication) with many other species currently being considered as well. One alternative for researchers working with organisms that do not have a reference genome is to use the reference genomes of closely related species to design cross-species comparisons, such as the study by Fontanesi et al. (2011) in sheep and goat (Fontanesi et al., 2010). Taking advantage of the phylogenetic proximity of goats and sheep to cattle (Kijas et al., 2006) they designed a custom tiling-array-based on sequence from the Btau 4.0 cattle assembly. While this method is not ideal for detecting novel insertions in each respective organism, they did identify 177 CNVRs (~10.8 Mbp) and 127 CNVRs (~11.47 Mbp) in sheep and goat, respectively (Fontanesi et al., 2010, 2011). They noted that only 0.4% of the genome of both goat and sheep was predicted to be variable using this method, which is a significant underestimation compared to a similar study using array CGH that identified 28.1 Mbp (~1% of the genome) of variable sequence in the cattle genome (Liu et al., 2010). Fontanesi et al. (2011) attribute any loss of accuracy in their method to poor DNA hybridization to several large sections of the cross-species tiling array that they had developed. While it bypasses the direct need for a reference assembly, this method still requires extensive molecular validation in order to achieve any degree of confidence in comparative CNV calls.

### THE GENOME ANNOTATION PROBLEM

Genome annotation also represents a substantial problem, as our means to sequence individual animals has greatly outpaced our ability to infer functional information from genetic sequence. There are currently 47,433 RefSeq transcripts in the GHCR37 genome assembly, which is substantially higher when compared to the number of annotated RefSeq transcripts present in cattle (14,176; UMD3.1), sheep (828; oviAri3), chicken (6501; galGal4), and pig (4921; susScr3). The current number of annotated transcripts in livestock genomes reflects a poor quality of assembly annotation rather than a genuine loss of gene number in these species. Recent initiatives from human researchers such as the ENCODE (Consortium, 2012) and GENCODE (Harrow et al., 2012) projects have begun to tackle the issues surrounding genome annotation by using experimental evidence to refine gene models and genomic functional regions. Predictions that over 93% of the human genome is transcribed (Consortium, 2012) and that there are nearly 10,000 human pseudogenes (Harrow et al., 2012). These facts suggest that proper genome annotation would be of great assistance in linking an organisms’ genotype to observed phenotypes. While the sheer-scale of such projects makes their reproduction for every livestock species difficult, there exists the possibility that functional elements discovered through the ENCODE project could be used in cross-species homology searches against existing livestock genomes. Even still, such comparisons will need to be conservative. Given the current constraints on the in-silico identification of transcription factor binding sites (Struckmann et al., 2011) and the limited number of genomic regions that are highly conserved among eutherian mammals (Lindblad-Toh et al., 2011), functional prediction from cross-species sequence homology is stymied by accumulated mutations after species divergence. New efforts devoted to functional sequence prediction in food animals, such as the AGENCODE project (Silverstein, J., personal communication), seek to emulate the human model and will provide excellent starting material for the community.

In addition to gene and functional element annotation, the identification of SDs within each genome provides predictive power toward the characterization of CNVs in individuals. Studies in cattle (Bickhart et al., 2012), dog (Nicholas et al., 2009), and pig (Paudel et al., 2013) have identified a 65.7%, 20%, and 27.5% overlap of SDs with CNVs, respectively. In each instance, the association of CNVs and SD regions was found to be statistically significant, as has been previously reported in human studies (Sharp et al., 2005; Alkan et al., 2009). Given that SD regions are prone to expansion due to NAHR of sister chromosomes across SD regions (Kim et al., 2008), their presence provides crucial data for the discovery of variable regions in the host genome. Unfortunately, the variable and repetitive nature of SDs makes them problematic to detect. A large proportion of SDs have been discovered in the unplaced contigs of the cattle (Liu et al., 2009) genome. This finding illustrates a significant problem, as the unplaced contigs represent difficult to assemble regions of the reference. An enrichment of SDs in these contigs could be a symptom of assembly difficulties, which in turn result in a loss of functional information that could otherwise be used to identify CNV hotspots in the genome. Additionally, misassembled contigs that have been placed on chromosomes in a reference assembly can be mistaken as false positive SD regions, as a recent comparison of two different cattle assemblies found that 39 Mbp of previously identified SDs were likely assembly errors and were not true SDs (Zimin et al., 2012).

### DIFFERENT KARYOTYPES AND THEIR IMPLICATIONS ON SV IDENTIFICATION

Chromosome fusions and translocations are balanced SVs that are also difficult to track using conventional genotyping platforms. Identification of the KIT gene translocation in Belgian Blue cattle (Durkin et al., 2012) reinforces the fact that chromosome translocations also impact organism phenotype; however, the difficulty in detecting these events may have resulted in an underestimation of their presence and effect. Similar to how a chromosome fusion can cause polyploidy via subsequent inheritance, translocation of a gene can also increase the copy number of that gene when two wild-type copies are inherited from the original chromosome, and the translocated copy is inherited as well.

Inversions and balanced chromosomal translocations are difficult to detect and more work needs to be done to track them. Inversions remain difficult to detect and validate due to the frequency of false positive signals from paired-end discordancy (also called “read pair” or RP) analysis algorithms (Korbel et al., 2007). While RP methods should provide a suitable means for detecting such events in theory, two major problems currently challenge the accuracy of this method: (1) alignment errors resulting from the mapping of read pairs to repetitive regions of the genome, and (2) the creation of duplicated, chimeric sequence fragments during the creation of paired-end libraries (Quail et al., 2008). The second problem (2) can be resolved through the use of strict sequence data quality controls that remove optical duplicate read fragments from consideration in addition to the use of strict data filters that require a high count of supporting discordant reads before inversions are called. This strategy is quite effective at removing potential sources of false positive data because it can be assumed that the chimeric read fragments will be rare species generated during library preparation, and that their influence on the final data is dependent on biased PCR amplification. Therefore, suitable quality control can reduce or eliminate chimeric signals from interfering with balanced SV detection. The first problem (1) is unfortunately dependent on the reference genome assembly for the species, and is unlikely to be resolved until better reference assemblies are created for livestock. Even still, some regions of the genome have similar repetitive regions close to each other, which would still impede accurate detection of inversions. This is quite vexing, as such regions are most likely to harbor insertions due to NAHR of the flanking repeats (Gu et al., 2008).

## FUTURE DIRECTIONS

### ROUTINELY TRACKING SVs WITHIN THE GENOME

While a large percentage of genomic variation among individuals is comprised by SVs (Redon et al., 2006), significant barriers exist that prevent them from being routinely tracked in genomic evaluations. As previously mentioned, the rate of *de novo* CNV generation (Itsara et al., 2010) is substantially different from that of single-nucleotide polymorphisms (SNPs; Kong et al., 2012). Additionally, duplication CNVs are particularly difficult to detect using array-based methods (Xu et al., 2013). Deletions appear to not only be easier to detect with a variety of different algorithms (Wang et al., 2007; Korn et al., 2008), but result in simpler haplotypes that may be easier to track and phase in affected individuals. While clear evidence exists that duplications play a major role in livestock phenotypes (Johansson Moller et al., 1996; Norris and Whan, 2008), the ease of which deletions can be predicted from existing datasets using array-based methods makes them a far more palatable target as markers for selection. In addition, deletions of DNA within genic regions may be the causative variants for disease phenotypes such as bovine anhidrotic ecotodermal dysplasia (Drögemüller et al., 2001), making the use of specific deletions as genetic markers a priority for the field.

**FIGURE 3:**
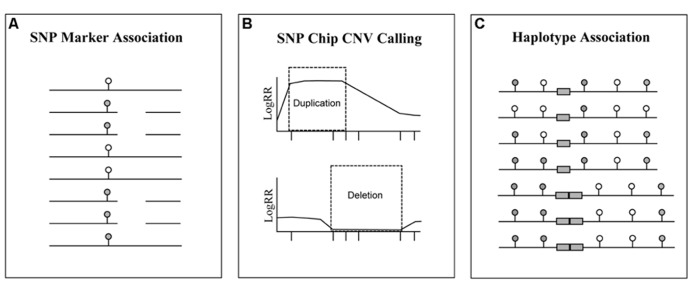
** Methods that can be used to track structural variants using genotyping platforms**. The ubiquity of SNP chip data for livestock species allows researchers the opportunity to track genomic segments with relative ease. Regardless, the association of SVs with SNP markers has proven to be problematic. Here are three strategies for tracking SVs using SNP genotyping arrays: **(A)** association of SNP marker genotypes (filled and empty ovals) with the SV; **(B)** identification of SVs from the logR ratio (LogRR) intensity of SNP probes (X axis tick marks); **(C)** and association of SVs (duplicated gray boxes) with SNP markers that form haplotypes.

Duplication SVs could be used as markers for livestock traits and diseases; however, methods to track them need to improve substantially (**Figure 3**). Existing methods use *a posteriori* information gleaned from the intensity of oligonucleotide probes on SNP genotyping platforms (Wang et al., 2007; Korn et al., 2008); however, the CNV calls from these tools suffer from high false discovery rates due to problems with SNP probe distance, experimental design and several other factors (Pinto et al., 2011; Xu et al., 2013). Given that the ubiquity of SNP genotype data in livestock species makes these methods attractive despite their high error rates, the potential to still use SNP genotypes to track SVs is tempting. One alternative is to use the knowledge of existing SV locations as a priori information, and to track SNP array-derived haplotypes (Browning and Browning, 2007; VanRaden et al., 2011) that overlap the known SV locations across individuals as was done by Boettger et al. (2012) for CNVs within the 17q21.31 region in humans. One problem with this approach is that it requires knowledge of the breakpoints and copy number state of the SVs to be tracked within the population. Additionally, the possibility exists that CNVs common to several breeds of livestock animals may be transmitted on different haplotypes within separate populations. Still, current studies in human have shown that even low frequency CNVs can be associated with SNP haplotypes (McCarroll et al., 2008), suggesting that this strategy will be effective when refined haplotypes are developed for livestock species.

### IMPROVING THE REFERENCE ASSEMBLY

Continuing work on the human reference genome assembly has been performed since the draft assembly was generated in the year 2000 (Lander et al., 2001; Venter et al., 2001). Misassembled contigs and structural variants (SVs) have been resolved in each successive draft assembly, resulting in the 37th iteration serving as the best, current human reference assembly released to the public. By contrast, livestock reference assemblies have received far less attention and less reassembly work, with the cattle (Zimin et al., 2009), sheep (International Sheep Genomics Consortium et al., 2010), chicken (Burt, 2005), and pig (Groenen et al., 2012) assemblies currently at their sixth, first, fourth, and tenth iterations. Some livestock species, such as goats, have only recently been sequenced to generate a reference assembly (Dong et al., 2013). Most algorithms designed to identify CNVs only work based on comparisons to a reference genome sequence, making a reference assembly a high priority for the species that currently lack one.

The development of new methods to assist in *de novo* assembly may promote the generation of new reference genomes for livestock species, or enhance existing assemblies. The goat assembly project initially used typical *de novo* assembly techniques involving the SOAPde novo software package (Luo et al., 2012), but also utilized an optical mapping technique in order to bypass the need for a physical genome map for scaffold placement (Dong et al., 2013). Optical mapping utilizes advances in molecule imaging technologies in order to visually identify DNA sequence features (i.e., restriction sites or fluorescent tags) in sequential fashion along a large DNA molecule [for a review please see: (Teague et al., 2010; Neely et al., 2011)]. While the resulting “sequence” derived from optical mapping is small (only the locations of restriction sites or specified tag sequences are known), the ability for the technology to span large segments of the genome allows it to anchor contigs and scaffolds that are generated from the use of shorter reads in *de novo* assembly. The technique is sufficient to resolve regions of the genome that are normally difficult to assemble due to their repetitive or copy number variable nature, such as the MHC locus assembly in the goat genome project (Dong et al., 2013). Recent efforts have been made to save information from sequence library creation to assist in assembly algorithms. Moleculo library creation is a technique that has been recently licensed by Illumina that generates longer read fragments from existing short read shotgun sequencing strategies. This technique was recently applied to *Botryllus schlosseri*, the colonial chordate, as a proof of concept study (Voskoboynik et al., 2013). The authors used a barcoding strategy to individually label larger strands of DNA generated from genomic shearing prior to library creation (Voskoboynik et al., 2013). Since the origins of the smaller sequence read fragments were maintained by the initial barcode, Voskoboynik et al. (2013) were able to assemble reads derived from each respective barcode type individually, thereby simulating larger read fragments on the order of 6–8 kbp in size.

Having been promoted for its longer read length compared to existing sequencing technologies, the Pacific Biosystems sequencer (PacBio) has been championed as a means by which researchers can close assembly gaps and repair assembly errors. English et al. (2012) used sequence data derived from the PacBio instrument to close gaps on a simulated *D. melanogaster* genome, the draft *D. pseudoobscura* assembly, the budgerigar assembly and the preliminary assembly of the Sooty mangabey. Results were promising, with 69%, 20%, 66% of all gaps in the *D. pseudoobscura*, budgerigar and mangabey assemblies, respectively, being closed by the longer read alignments (English et al., 2012). Still, the authors note that the high error rate (~15%; primarily comprised of artificial single nucleotide insertions) of the PacBio sequencer (Carneiro et al., 2012) necessitates a larger coverage of the genome in order to ensure accurate closure of assembly gaps. This can be inferred from their results as the budgerigar genome (4 X coverage) genome had fewer gap closures than *D. pseudoobscura* (24 X coverage) and mangabey (6.8 X coverage; English et al., 2012). Still, if a suitable coverage of longer reads can be achieved, gap closure in existing reference assemblies could be possible with PacBio data.

### USING BETTER METHODS AND IMPROVED TECHNOLOGY

SV detection resulting from the analysis of high throughput sequencing data appears to be the new gold standard by which the research community should proceed. Lower false discovery rates for several NGS-based algorithms (Abyzov et al., 2011; Handsaker et al., 2011), and their high concordance with validation assays (Mills et al., 2011) within the human 1000 genomes project provide ample support for the use of NGS data to create high quality CNV maps. Already, livestock researchers have started creating comprehensive NGS-based CNV maps in cattle (Stothard et al., 2011; Bickhart et al., 2012), chicken (Kerstens et al., 2011), and pig (Paudel et al., 2013). While sequencing costs still remain high enough to prevent the scanning of large populations of animals, such studies will need to be performed in the future in order to estimate CNV frequencies within animal populations. If CNV frequencies can be derived from population-based studies, potential *de novo* events can be detected with greater precision. Additionally, livestock species that are currently improved through selective breeding efforts often have extensive pedigrees. The development of methods that use pedigrees to improve CNV calling methods would be a great benefit to the research community. Finally, the development and constant maintenance of SD maps for each reference genome would provide useful context for the detection of CNVS. Given that NAHR among SDs is responsible for many unbalanced CNVs (Kim et al., 2008), knowledge of SD regions in a genome would allow researchers to estimate the likelihood of true CNV events based on SD proximity and the methods used to detect the CNVs. SD maps already exist for popular reference assemblies of the cattle (Liu et al., 2009; Zimin et al., 2012), chicken (Wang et al., 2010), sheep and pig (Groenen et al., 2012) genomes. The relative novelty of the field should be an encouragement to other researchers looking to expand these existing efforts with new contributions.

## AUTHOR CONTRIBUTIONS

Derek M. Bickhart and George E. Liu assembled relevant literature and wrote the manuscript.

## Conflict of Interest Statement

The authors declare that the research was conducted in the absence of any commercial or financial relationships that could be construed as a potential conflict of interest.
